# Post-traumatic headache as a window into persistent brain dysfunction after traumatic brain injury: an opinion article

**DOI:** 10.3389/fnhum.2026.1946999

**Published:** 2026-10-15

**Authors:** Denise Franco de Almeida Duarte, Jader Vinicius da Silva Rocha, Fernanda Moura Vargas Dias, Micheline Henrique Araújo da Luz Koerich, Vitória Caroline Reinoso de Almeida, Rodrigo Freire de Almeida, Walter Gomes Da Silva Filho, Fernando Zanela Da Silva Arêas

**Affiliations:** 1 Department of Physioligc Sciences, Neurorehabilitation and Neuromodulation Laboratory, Federal University of Espírito Santo, Vitória, Brazil; 2 Department of Physioterapy, Graduate Program in Physiotherapy Santa Catarina State University, Florianópolis, Brazil; 3 Baylor Scott & White Research Institute, Dallas, TX, United States

**Keywords:** biomarkers, brain connectivity, central sensitization, neuroinflammation, post-traumatic headache, traumatic brain injury

## Introduction

Traumatic brain injury (TBI) remains a major cause of death and disability worldwide, particularly among young adults and individuals of working age (Arêas et al., 2019; Andersen et al., 2020; Gonçalves et al., 2025). Advances in prehospital care, neurosurgery, and intensive care have improved survival after TBI, increasing attention to the persistent symptoms and functional limitations that may affect long-term recovery and quality of life (Arêas et al., 2019; Gonçalves et al., 2025).

Post-traumatic headache (PTH) is a secondary headache attributed to traumatic injury to the head, including TBI, and is among the most frequent neurological symptoms reported after TBI (Headache Classification Committee of the International Headache Society (IHS), 2018; Ashina et al., 2021; Labastida-Ramírez et al., 2020; Mavroudis et al., 2023). In this article, we specifically address PTH occurring in the context of TBI rather than the broader category of headache attributed to head injury without established TBI. Consistent with the International Classification of Headache Disorders, PTH that persists for more than 3 months is referred to here as persistent PTH [Headache Classification Committee of the International Headache Society (IHS), 2018]. Persistent PTH may coexist with fatigue, cognitive complaints, sleep disturbances, anxiety, depression, and limitations in daily activities (Ashina et al., 2021; Labastida-Ramírez et al., 2020; Mavroudis et al., 2023), contributing to the complexity of long-term clinical recovery.

Conventional measures of initial TBI severity, including the Glasgow Coma Scale (GCS), pupillary reactivity, and structural imaging findings, remain essential for acute assessment and prognosis. However, the relationship between the extent and characteristics of the initial injury and persistent symptoms is not straightforward, and individuals with apparently similar acute injury characteristics may follow different long-term recovery trajectories (Ashina et al., 2021; Labastida-Ramírez et al., 2020; Mavroudis et al., 2023; Brett et al., 2023; Nelson et al., 2023). This variability has increased interest in biological and functional processes that may persist or evolve after the acute injury (Simon et al., 2017; Witcher et al., 2021; Freire et al., 2023).

Against this background, we propose the hypothesis that persistent PTH may serve as a clinical window into persistent brain dysfunction after TBI. This concept does not imply that persistent PTH is itself a biomarker or that it demonstrates the presence of a specific underlying mechanism. Rather, we propose that its persistence may serve as a clinical signal warranting investigation of functional or biological abnormalities that are not fully characterized by conventional structural assessment.

This hypothesis is considered across the TBI severity spectrum. However, the evidence discussed in this article derives from heterogeneous populations, including mild TBI and moderate-to-severe TBI, and findings from one severity group should not be assumed to generalize to another. Furthermore, evidence directly obtained from patients with persistent PTH remains more limited than evidence derived from broader TBI populations. Throughout this article, we therefore distinguish PTH-specific findings from mechanisms that remain biologically plausible on the basis of the broader TBI literature.

## Beyond structural injury: persistent brain dysfunction after TBI

Acute TBI severity remains a major determinant of early clinical outcomes. Measures such as the GCS, pupillary reactivity, and structural imaging findings are strongly associated with mortality and functional disability and remain fundamental for clinical decision-making and prognostic assessment (Arêas et al., 2019; Gonçalves et al., 2025; Bertotti et al., 2023). However, these measures were primarily developed to characterize the initial injury and estimate acute outcomes, and their ability to account for persistent symptoms and heterogeneous long-term recovery trajectories is more limited (Brett et al., 2023; Nelson et al., 2023).

Patients with apparently similar initial injury characteristics may experience markedly different long-term outcomes, including persistent headache, fatigue, cognitive impairment, sleep disturbances, emotional symptoms, and reduced quality of life (Brett et al., 2023; Nelson et al., 2023). This variability suggests that the extent and characteristics of the initial injury alone do not fully capture the processes associated with long-term recovery.

TBI initiates a cascade of biological events that may extend beyond the acute phase (Simon et al., 2017; Witcher et al., 2021; Freire et al., 2023). Among these processes, neuroinflammation has been extensively investigated. Acute activation of microglia and astrocytes contributes to tissue responses following injury, whereas sustained or dysregulated inflammatory activity has been associated with blood–brain barrier alterations, persistent inflammatory signaling, and changes in neuronal function (Simon et al., 2017; Witcher et al., 2021; Freire et al., 2023; Loane and Kumar, 2016; Henry et al., 2020; Faden and Loane, 2015). Importantly, evidence of persistent neuroinflammation after TBI derives predominantly from experimental studies and broader TBI populations and should not be interpreted as evidence that neuroinflammation directly causes persistent PTH. Rather, it provides a biologically plausible framework that requires specific validation in patients with persistent PTH.

Altered nociceptive processing may represent another relevant mechanism. Central sensitization should be distinguished from nociplastic pain and other forms of altered nociceptive signaling because these concepts are related but not interchangeable (Nijs et al., 2021; Woolf, 2011). PTH-specific studies have identified sensory abnormalities, including cephalic allodynia, hyperalgesia, and altered pain modulatory responses, supporting abnormal nociceptive processing in at least a subset of patients with persistent PTH (Defrin et al., 2010). Experimental evidence of hypersensitivity to calcitonin gene-related peptide (CGRP) further supports the involvement of CGRP-related nociceptive pathways (Ashina et al., 2020). However, these findings neither establish central sensitization as a universal mechanism nor demonstrate a single pathophysiological pathway underlying persistent PTH.

A complementary perspective is provided by network neuroscience, which conceptualizes neurological dysfunction as potentially arising from disturbances in interactions among distributed brain networks rather than exclusively from focal structural damage (Bassett and Sporns, 2017; Crossley et al., 2014; Menon, 2011). TBI studies have demonstrated persistent structural network abnormalities during recovery (Gugger et al., 2023), while PTH-specific neuroimaging studies have identified alterations in resting-state functional connectivity involving pain-processing, pain-modulatory, and affective networks (Niu et al., 2019; Dumkrieger et al., 2019). These findings provide more direct, although still limited, evidence that persistent PTH may be associated with alterations in brain network function.

Persistent PTH also frequently co-occurs with fatigue, cognitive impairment, sleep disturbances, emotional symptoms, and reduced quality of life (Ashina et al., 2021; Labastida-Ramírez et al., 2020; Mavroudis et al., 2023). Rather than representing different expressions of a single persistent biological condition, these manifestations may arise from partially overlapping, independent, or mutually reinforcing mechanisms. Their co-occurrence therefore underscores the heterogeneity of post-traumatic recovery and the need to investigate how different biological and functional processes contribute to individual symptom profiles.

## Post-traumatic headache as a clinical window into persistent brain dysfunction

Within the framework proposed here, persistent PTH is considered a potential clinical window into persistent brain dysfunction rather than a validated biomarker, prognostic indicator, or surrogate measure of an underlying biological process. We use the term clinical window to describe an observable clinical feature whose persistence may signal the need to investigate ongoing functional or biological abnormalities that are not fully captured by conventional structural assessment. Thus, persistent PTH is proposed primarily as a clinical signal for further investigation, not as evidence that a specific pathophysiological process is present.

We use the term persistent brain dysfunction as an umbrella concept encompassing ongoing abnormalities in neural function or biological processes after TBI, including altered nociceptive processing, disturbances in brain network function, and potentially persistent inflammatory activity. This definition does not imply a single mechanism, nor does it assume that these abnormalities are present in all individuals with persistent PTH or that persistent PTH is caused by any one of them.

The distinction is important because the evidence supporting these potential mechanisms varies substantially. PTH-specific neuroimaging studies have demonstrated alterations in resting-state functional connectivity involving pain-processing and pain-modulatory networks, including periaqueductal gray-related connectivity, with some alterations associated with headache burden or the subsequent development of persistent PTH (Niu et al., 2019; Dumkrieger et al., 2019). By contrast, evidence for persistent neuroinflammation and several other chronic neurobiological abnormalities derives predominantly from broader TBI populations. These mechanisms should therefore be regarded as biologically plausible contributors requiring direct investigation in persistent PTH rather than as established pathophysiological correlates of the condition.

Persistent PTH may also reflect greater overall symptom burden rather than a distinct biological state. Headache commonly co-occurs with sleep disturbance, fatigue, cognitive complaints, anxiety, depression, and other post-traumatic symptoms (Ashina et al., 2021; Labastida-Ramírez et al., 2020; Mavroudis et al., 2023), and these factors may independently influence both headache persistence and measures of brain function. Pre-existing headache disorders, injury severity, medication use, cervical or extracranial pain generators, and psychological and behavioral factors may further contribute to clinical heterogeneity. Consequently, the presence or severity of persistent PTH cannot currently be assumed to indicate the presence, type, or magnitude of persistent brain dysfunction.

Testing the proposed clinical-window hypothesis will therefore require prospective studies that combine detailed headache phenotyping with objective measures of brain function and biology while accounting for relevant confounding factors. Quantitative electroencephalography, neuroimaging-derived connectivity measures, and molecular or inflammatory measures are candidate approaches that could be evaluated in this context (Gugger et al., 2023; Johnson et al., 2022; Huie et al., 2021; Thatcher et al., 2001). Artificial intelligence and machine-learning methods may complement these measures as analytical approaches for identifying multidimensional patterns or patient phenotypes, rather than representing biological or functional measures themselves (Folweiler et al., 2020; Cao et al., 2008).

At present, none of these candidate approaches can be considered a validated biomarker of persistent PTH. Their value lies in testing whether persistent PTH is systematically associated with measurable patterns of post-traumatic dysfunction beyond headache severity and overall symptom burden.

## Discussion

The hypothesis proposed in this article should be interpreted within several important limitations. First, evidence directly linking persistent PTH to measurable ongoing brain dysfunction remains limited. Although PTH-specific neuroimaging studies have identified alterations in functional connectivity (Niu et al., 2019; Dumkrieger et al., 2019), much of the evidence supporting persistent neuroinflammation, neurophysiological abnormalities, and other chronic biological processes derives from broader TBI populations. Extrapolation of these findings to persistent PTH therefore requires caution, and associations observed after TBI should not be assumed to represent mechanisms of persistent PTH.

Second, persistent PTH is clinically and biologically heterogeneous. Headache phenotype, pre-existing headache disorders, TBI severity, sleep disturbance, fatigue, psychological symptoms, medication use, cervical or extracranial pain generators, and other post-traumatic symptoms may influence both headache persistence and measures of brain function. These factors provide alternative or complementary explanations for associations between persistent PTH and biological or functional abnormalities. Consequently, future studies should determine whether such associations remain after appropriate characterization and adjustment for relevant confounding factors.

The proposed clinical-window hypothesis also generates testable predictions. If persistent PTH is associated with ongoing post-traumatic dysfunction beyond overall symptom burden, individuals with persistent PTH should demonstrate reproducible functional or biological abnormalities when compared with appropriately matched TBI survivors without persistent PTH. These associations should remain detectable after accounting for injury severity, pre-existing headache, headache phenotype, psychological symptoms, sleep disturbance, medication use, and other relevant clinical factors. Longitudinal studies should further determine whether changes in headache frequency, intensity, or disability are accompanied by changes in candidate neurophysiological, neuroimaging, or molecular measures.

Conversely, failure to identify reproducible associations between persistent PTH and objective measures of post-traumatic dysfunction, particularly after controlling for symptom burden and relevant confounders, would argue against the proposed clinical-window hypothesis or restrict its applicability to specific patient subgroups. This possibility is important because the framework proposed here should be considered a testable hypothesis rather than an assumption that persistent headache necessarily reflects ongoing brain dysfunction.

Future research should therefore prioritize prospective longitudinal designs with detailed headache phenotyping and appropriate comparison groups. Studies comparing TBI survivors with persistent PTH, TBI survivors without persistent PTH, and, where appropriate, individuals with primary headache disorders may help determine which findings are related specifically to persistent PTH, to TBI more broadly, or to chronic headache irrespective of trauma. Stratification by mild TBI and moderate-to-severe TBI will also be necessary because mechanisms and recovery trajectories may differ across injury severity.

Multimodal studies integrating clinical phenotyping with neuroimaging, quantitative electroencephalography, and molecular measures may help determine whether reproducible patterns of dysfunction can be identified in specific persistent PTH subgroups. Artificial intelligence and machine-learning approaches may support the analysis of these multidimensional datasets and the identification of patient phenotypes, but their role is analytical and depends on the quality and validation of the underlying clinical and biological measures (Gugger et al., 2023; Johnson et al., 2022; Huie et al., 2021; Folweiler et al., 2020; Cao et al., 2008; Thatcher et al., 2001). Candidate measures should not be considered biomarkers until their reproducibility, specificity, longitudinal behavior, and clinical relevance have been established in persistent PTH populations.

Ultimately, the value of the proposed framework will depend on whether persistent PTH provides clinically meaningful information beyond established measures of injury severity and overall post-traumatic symptom burden. Prospective validation is therefore required before persistent PTH can be used to identify individuals for additional biological assessment, prognostic stratification, or mechanism-based interventions.

Persistent PTH may provide a clinically accessible signal for investigating ongoing functional or biological abnormalities after TBI, but this relationship remains hypothetical and should not be interpreted as evidence that persistent headache is itself a biomarker of brain dysfunction. Distinguishing PTH-specific findings from abnormalities associated with TBI more broadly, controlling for alternative explanations and confounding factors, and prospectively testing the proposed associations will be essential to determine the validity and clinical relevance of this framework.
